# Impact of Sacubitril/Valsartan (ARNI) Compared with ACEI/ARB in Patients with Acute Myocardial Infarction on Post-Infarction Left Ventricular Systolic Dysfunction: A Retrospective Analysis

**DOI:** 10.3390/biomedicines13092265

**Published:** 2025-09-15

**Authors:** Rafał Niemiec, Małgorzata Niemiec, Martyna Nowak, Barbara Gurba, Monika Bujak, Katarzyna Chowaniec-Rybka, Magdalena Sowier, Agnieszka Nowotarska, Bartosz Gruchlik, Adam Pytlewski, Katarzyna Mizia-Stec

**Affiliations:** 1 First Department of Cardiology, School of Medicine in Katowice, Upper Silesian Medical Centre, Medical University of Silesia, 40-055 Katowice, Poland; 2 Students’ Scientific Club of First Department of Cardiology, School of Medicine in Katowice, Upper Silesian Medical Centre, Medical University of Silesia, 40-055 Katowice, Poland; 3 Faculty of Electrical and Computer Engineering, Cracow University of Technology, 31-155 Cracow, Poland

**Keywords:** ARNI, AMI, ACEI/ARB, angiotensin receptor–neprilysin inhibitor, acute myocardial infarction, post-infarction left ventricular systolic dysfunction

## Abstract

**Background/Objectives**: Angiotensin receptor–neprilysin inhibitor (ARNI) has a well-established advantage over angiotensin-converting enzyme inhibitor or angiotensin receptor blocker (ACEI/ARB) therapy in patients (pts) with heart failure with reduced ejection fraction (HFrEF), but in pts after acute myocardial infarction (AMI) with left ventricular (LV) systolic dysfunction, the advantage of ARNI has not been clearly proven. The efficacy of ARNI is compared with that of ACEI/ARB therapy in patients with their first AMI in terms of improvement of post-infarction LV systolic function. **Methods**: The study was conducted as a retrospective one-center cross-sectional analysis. Overall, 1473 pts (990 M, median age 71 [64; 77]) with AMI (their first AMI, complete coronary revascularization, no prior coronary revascularization or history of HF) hospitalized in 2022–2024 were enrolled in a retrospective cross-sectional analysis. The study population was categorized into pts receiving ARNI and ACEI/ARB. Then, based on the ARNI subgroup, matching that included age, sex, and LV ejection fraction (LVEF) was performed by using the 1:1 nearest neighbor method without returning. Finally, two groups (ARNI vs. ACEI/ARB) of 30 pts were obtained and analyzed at baseline and at a 6-week follow-up. The improvement of post-infarction LV systolic function was obtained in terms of LVEF, ΔLVEF, and relative ΔLVEF values (ΔLVEF/baseline LVEF). **Results**: The comparison of baseline characteristics revealed borderline lower initial LVEF (30 vs. 36%, *p* = 0.076) and a higher frequency of SGLT-2 inhibitor use (70% vs. 36.7%, *p* = 0.01) in the ARNI subgroup. At the 6-week follow-up, in both subgroups, a significant improvement in the median LVEF values was achieved—from a median LVEF value of 30% (27.3; 38) to 37% (30; 43; *p* = 0.0008) in the ARNI subgroup and from a median LVEF value of 36% (33; 39) to 45% (42; 52; *p* < 0.0001) in the ACEI/ARB subgroup. The median ΔLVEF in the ACEI/ARB subgroup was higher [10% (6; 12)] than in the ARNI subgroup [6% (2; 10.25), *p* = 0.018]. Similarly, the median relative ΔLVEF was higher in the ACEI/ARB subgroup [30% (15.4; 40)] than in the ARNI group [17.5% (7; 31.9), *p* = 0.047]. The vast majority of patients, particularly in the ARNI group (99.7%), were treated with the lowest available dose of the drug. **Conclusions**: Our current experience in ARNI therapy after AMI is promising; however, it is limited to a small group of patients with severe impairment of LV systolic function. Regardless of the significant improvement in the baseline LVEF observed in patients receiving both ACEI/ARB and ARNI at the 6-week follow-up, the absolute and relative increases in the LVEF were higher in subjects treated with ACEI/ARB. However, the clinical benefits of ARNI therapy may emerge more gradually, and its advantages could become more apparent over a longer follow-up period. The clinical efficacy of early use of ARNI in the setting of AMI needs further evaluation.

## 1. Introduction

The use of angiotensin receptor–neprilysin inhibitor (ARNI) in patients after acute myocardial infarction (AMI) remains an area of intense clinical research and debate. The data to date clearly support the benefits of sacubitril/valsartan in patients with heart failure with reduced ejection fraction (HFrEF), but its efficacy in the post-myocardial infarction (MI) population with left ventricular systolic dysfunction has not yet been unequivocally proven.

The landmark PARADIGM-HF trial demonstrated that ARNI was superior to enalapril in reducing the risk of cardiovascular death and hospitalization for heart failure, leading to a change in guidelines that made it the preferred therapy for patients with HFrEF [1,2,3]. This raised the question of whether even earlier administration of ARNI initiated soon after MI in patients with impaired LVEF but not yet with symptomatic heart failure could offer additional benefits in preventing remodeling and improving survival compared with conventional ACEI-based pharmacotherapy. The PARADISE-MI trial was designed to address this question; however, the primary outcome showed no statistically significant difference between the two groups in the composite endpoint of death from cardiovascular causes or incident heart failure (HF), whichever occurred first [4]. The PARADISE-MI Echocardiographic Substudy also failed to demonstrate significant differences between ARNI and ramipril in terms of changes in the left ventricular ejection fraction (LVEF) or left atrial volume (LAV) after 8 months of treatment [5].

In recent years, there have been many studies on the use of ARNI in MI. These studies are based on animal models, randomized clinical trials, systematic reviews, and meta-analyses. The results of these studies indicate the advantage of the use of ARNI over ACEI/ARB in patients with MI or at least suggest that ARNI use is non-inferior to conventional drug therapy. Additionally, there are many studies on animal models that indicate a number of benefits resulting from the use of ARNI, such as antiarrhythmic and anti-inflammatory effects on post-MI scarring, the amelioration of cardiac function and ventricular remodeling, and a myocardial protective effect.

In our clinical practice, we always try to make decisions based on the latest medical knowledge and with the best interests of our patients in mind. In this article, we present the results of a real-world study evaluating the use of ARNI in patients with AMI, with a particular focus on those with significantly reduced LVEF. What distinguishes this study is its emphasis on a real-life clinical setting combined with a data-matching methodology to ensure comparability between the treatment groups. Importantly, the analysis specifically targets patients with severely impaired left ventricular systolic function, providing valuable insight into a population that is often underrepresented in randomized trials. The aim of the study was to compare the efficacy of ARNI with that of ACEI/ARB therapy following a first AMI with regard to improvement in post-infarction left ventricular systolic function.

## 2. Materials and Methods

### 2.1. Data Collection and Study Design

The study was conducted as a retrospective one-center cross-sectional analysis of 1571 consecutive hospitalizations for AMI. In the analysis, we included patients with a diagnosis of AMI using the International Classification of Diseases (ICD) billing codes (10th edition, I21.X). The analysis comprised patients hospitalized in the I Department of Cardiology, the Medical University of Silesia, in Katowice, from January 2022 to December 2024.

Inclusion and exclusion criteria were a key part of this study. The study included patients aged 18 years or older with a first diagnosis of myocardial infarction who underwent percutaneous revascularization of the infarct-related coronary artery followed by complete revascularization during current hospitalization, had an LVEF of 40% or less, and were enrolled in coordinated specialist care after an MI (“KOS zawał” program).

Patients were excluded if they had a history of previous MI or coronary revascularization, a history of HF or angioedema, co-occurrence of another heart disease affecting the LVEF, severe hepatic or renal dysfunction, coexisting severe diseases of the immune, hematopoietic, or respiratory systems, systemic diseases, or presence of a malignant tumor; the exclusion criteria also included the death of the patient during hospitalization and the lack of complete clinical data in the medical records. A summary of the inclusion and exclusion criteria is presented in Table 1.

Among all 1571 hospitalizations, 1473 individual patients were identified, and their first hospitalization was analyzed. The study population was divided based on ARNI or ACEI/ARB administration and analyzed using the inclusion and exclusion criteria. In the first stage, after taking into account patient death, history of HF, previous MI or coronary revascularization, and lack of inclusion in the coordinated care program (KOS-zawał program), 1354 patients were excluded from the study. In this way, 119 patients were selected and further analyzed. In the second stage, upon application of additional criteria, such as incomplete revascularization during current hospitalization, incomplete clinical data in the medical records, malignant tumors in their medical history, qualification for coronary artery bypass grafting (CABG), and co-occurrence of another heart disease affecting the LVEF, an additional 43 patients were excluded, and the remaining 76 patients (30 treated with ARNI and 46 treated with ACEI/ARB) were included in detailed analysis and matching.

A total of 1397 (91.9%) patients were excluded from the initial cohort, and only 76 (5.2%) were considered for further analysis. The primary aim of the selection process was to construct a study population that most accurately reflects patients with left ventricular systolic dysfunction resulting solely from MI, ensuring that the observed LV dysfunction was not attributable to other causes. Additionally, patients with significant comorbidities were excluded, and the remaining criteria were carefully designed to isolate the effects of ARNI and ACEI/ARB therapy on the LVEF while minimizing the confounding influence of external factors on the outcome. All patients were compensated at discharge and received an optimal medical therapy of AMI and HFrEF, including dual antiplatelet therapy, lipid-lowering treatment, ACEIs/ARB (quinapril, enalapril, perindopril, ramipril, zofenopril/telmisartan, valsartan) or ARNI, mineralocorticoid-receptor antagonists (MRAs), beta-blockers, and diuretics in patients with symptoms and/or signs of congestion. The data were obtained from electronic medical records.

### 2.2. Matching

Taking into account differences in the sex, age, and LVEF between the ARNI and ACEI/ARB subgroups, matching was performed using the 1:1 nearest neighbor (NN) method without returning. The matching resulted in two groups, each with 30 patients where the mean age, LVEF, and gender proportions were more comparable between both. Subgroups of 30 patients were subjected to further analysis to compare the baseline characteristics and clinical outcomes.

### 2.3. Outcomes

The outcome analysis consisted of LVEF data obtained in 6 weeks and 4 months of follow-up (a limited number of patients) and cardiovascular outcomes (median follow-up: 695 [456; 996] days). During the 6-week follow-up period, ACEI/ARB and ARNI doses were not escalated.

The improvement of post-infarction left ventricular systolic function was obtained in terms of direct LVEF, ΔLVEF, and relative ΔLVEF values. ΔLVEF was defined as the final LVEF − baseline LVEF (where the final LVEF is understood as the LVEF after 6 weeks or 4 months of follow-up, and the baseline LVEF is understood as the LVEF at the diagnosis of MI). The relative ΔLVEF was defined as the percentage change from the baseline LVEF (percentage of the baseline LVEF). Relative ΔLVEF = (final LVEF − baseline LVEF)/baseline LVEF × 100%. The mathematical formulas for ΔLVEF and relative ΔLVEF are shown below.

ΔLVEF=final LVEF−baseline LVEF

$$relative\;\Delta LVEF=\frac{final\;LVEF-baseline\;LVEF}{baseline\;LVEF}\times 100\%$$

Echocardiographic examinations were performed by experienced echocardiographers, and the LVEF was assessed either visually or using the Simpson biplane method. During the assessments, the echocardiographers were blinded to the patient’s treatment allocation.

### 2.4. Statistical Analysis

The study population was first dichotomized into 2 groups: those receiving ARNI or ACEI/ARB. Clinical characteristics and outcomes were compared between groups. Continuous variables are presented as the mean ± standard deviation or median (1–3 quartiles), and the categorical variables are presented as absolute values and percentages. Normality was verified using the Shapiro–Wilk test. The comparisons of groups were based on Student’s two-sample *t*-tests, nonparametric Mann–Whitney U tests, or chi-squared test, as appropriate. The Wilcoxon signed-rank test or Student’s *t*-test for related variables was used to analyze related variables. A *p*-value of ≤0.05 was considered statistically significant for all tests. Matching was performed by using the 1:1 nearest neighbor (NN) method without returning—at first, the logistic regression model was trained on the study population; secondly, propensity scores were calculated for each patient. Finally, patients from the ARNI and ACEI/ARB subgroups were matched 1:1 using the nearest neighbors (NN) algorithm with the propensity score difference as a distance measure. All other analyses were performed using the MedCalc^®^ version 23.2.1 software.

## 3. Results

### 3.1. Study Population and Inclusion and Exclusion Criteria

**Figure 1:**
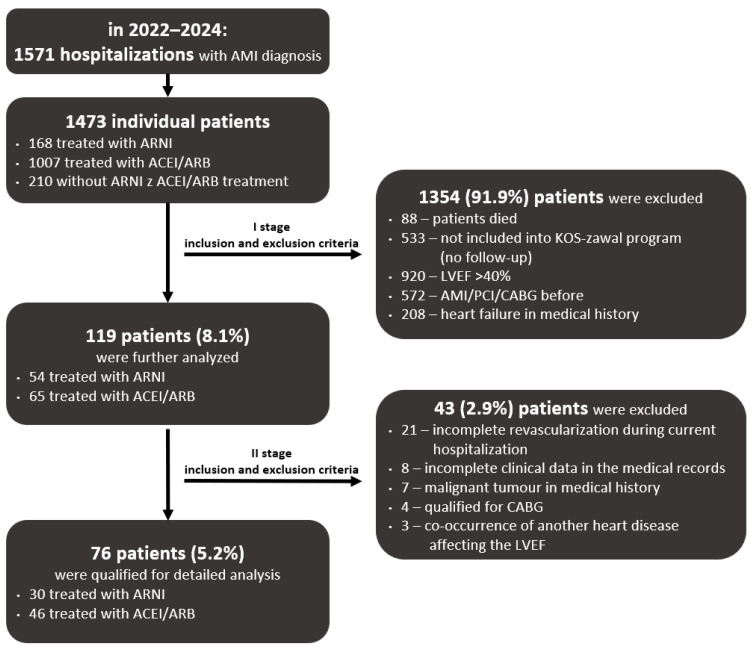
Detailed analysis of the inclusion and exclusion criteria. ACEI: angiotensin-converting enzyme inhibitor; AMI: acute myocardial infarction; ARB: angiotensin II receptor blocker; ARNI: angiotensin receptor–neprilysin inhibitor; CABG: coronary artery bypass grafting; LVEF: left ventricular ejection fraction; PCI: percutaneous coronary intervention.

In the years 2022–2024, there were 1571 hospitalizations due to AMI, of which 1473 individual patients were identified (990 men, median age 71 [64; 77]). After applying the inclusion and exclusion criteria in the first stage, 1354 (91.9%) patients were excluded from the study. In the second stage, the remaining 119 (8.1%) patients were further analyzed, and based on the criteria provided, an additional 43 (2.9%) patients were excluded. The remaining 76 patients (5.2%) underwent matching and detailed analysis. The details of the analysis of the inclusion and exclusion criteria are presented in Figure 1.

### 3.2. Matching Results

**Figure 2:**
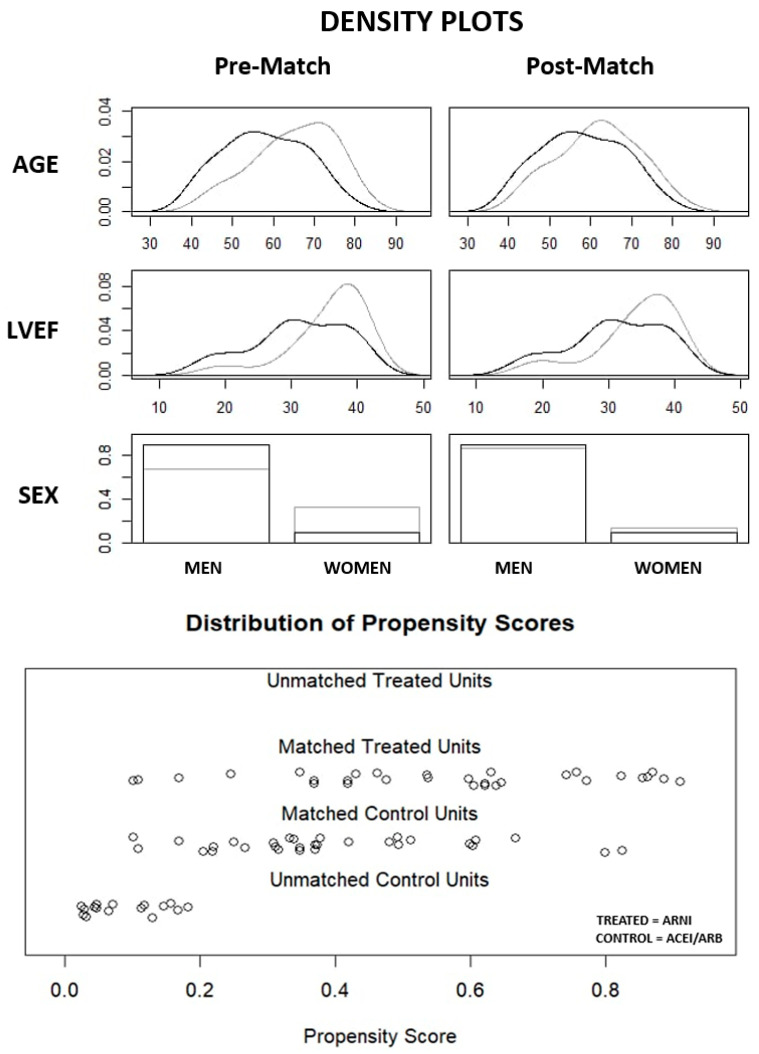
Density plots and distribution of propensity scores before and after matching. ACEI: angiotensin-converting enzyme inhibitor; ARB: angiotensin II receptor blocker; ARNI: angiotensin receptor–neprilysin inhibitor; LVEF: left ventricular ejection fraction.

The detailed analysis included 76 patients, among whom two subgroups were distinguished: the ARNI subgroup (39.5%; 27 men; age 58 ± 10 years) and the ACEI/ARB subgroup (60.5%; 31 men; age 65 ± 10 years). The compared values of the study population before and after matching are detailed in Table 2. Density plots and the distribution of propensity scores are presented in Figure 2.

### 3.3. Comparison of the Baseline Characteristics of the ARNI and ACEI/ARB Subgroups

The patients in the two subgroups did not differ clinically. In both subgroups, the mean age was about 60 years; 90% of the patients were men, and the average BMI was about 29 kg/m^2^. The vast majority of patients (about 80%) were diagnosed with ST-elevation myocardial infarction (STEMI). Additionally, no statistically significant differences were observed in basic laboratory results.

In the ARNI subgroup, patients had a lower initial LVEF (30 vs. 36%, *p* = 0.076), but the difference was not statistically relevant.

In terms of comorbidities, both groups were quite similar, with no substantial burden of multimorbidity. Most of them suffered from hypertension and hyperlipidemia and had a history of nicotine addiction. No statistically significant differences were observed between the subgroups.

Patients in both subgroups were treated in a similar way. The only difference was the rate of sodium-glucose co-transporter 2 (SGLT-2) inhibitor use, which was higher in the ARNI subgroup (70% vs. 36.7%, *p* = 0.01). Detailed baseline characteristics of patients in the ARNI and ACEI/ARB subgroups are presented in Table 3.

### 3.4. LVEF Improvement in 6 Weeks of Follow-Up in the ARNI and ACEI/ARB Subgroups

In the ARNI and ACEI/ARB subgroups, at the 6-week follow-up, a significant improvement in the LVEF was achieved. In the ARNI subgroup, the median improvement in the LVEF was from 30% (27.3; 38) to 37% (30; 43; *p* = 0.0008), and in the ACEI/ARB subgroup, the median LVEF improvement was from 36% (33; 39) to 45% (42; 52; *p* < 0.0001). The median ΔLVEF in the ACEI/ARB subgroup was higher [10% (6; 12)] than in the ARNI subgroup [6% (2; 10.25), *p* = 0.018]. Similarly, the median relative ΔLVEF was higher in the ACEI/ARB subgroup [30% (15.4; 40)] than in the ARNI group [17.5% (7; 31.9), *p* = 0.047]. Additionally, the direct value of the LVEF after 6 weeks was statistically significantly higher in the ACEI/ARB subgroup 45% (45; 52) than in the ARNI subgroup 37% (30; 43), *p* = 0.003.

### 3.5. LVEF Improvement in 4 Months of Follow-Up in the ARNI and ACEI/ARB Subgroups

At the 4-month follow-up, statistically significant improvements in the LVEF were also achieved in both subgroups—from 30.5 ± 5.9% to 40.2% ± 7.8 (*p* = 0.0001) in the ARNI subgroup and from 34.9 ± 7.3% to 44.6 ± 10.9% (*p* = 0.018) in the ACEI/ARB subgroup. The values of ΔLVEF and relative ΔLVEF were similar between the subgroups. The analysis at the 4-month follow-up is based on a small patient cohort; therefore, the interpretation of the results should consider the potential risk of a type II error.

**Figure 3:**
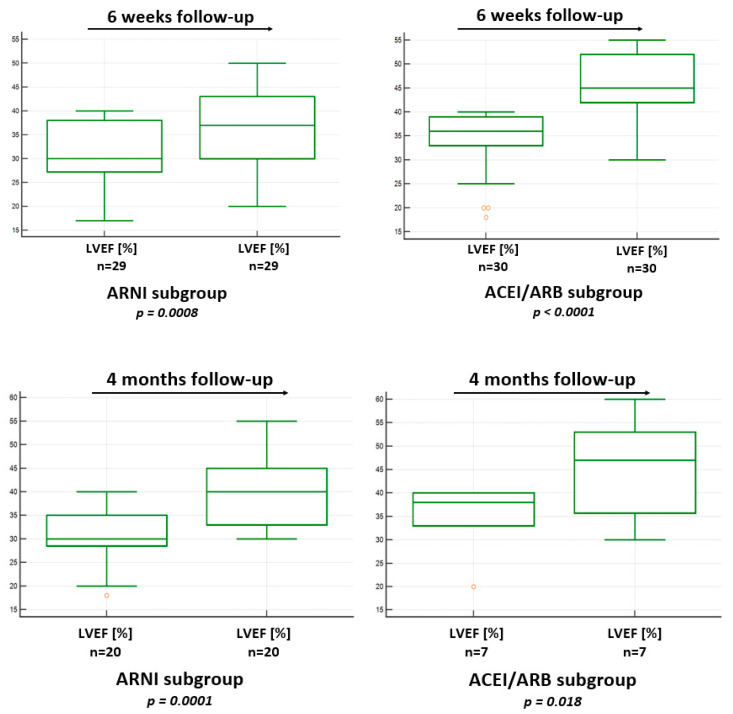
Comparison of LVEF improvements at the 6-weeksand 4-month follow-up in the ARNI and ACEI/ARB subgroups. ACEI: angiotensin-converting enzyme inhibitor; ARB: angiotensin II receptor blocker; ARNI: angiotensin receptor–neprilysin inhibitor; LVEF: left ventricular ejection fraction.

The results of the direct LVEF improvement at 6 weeks and 4 months of follow-up are presented in Figure 3 and Table 4 and Table 5. A detailed comparison of the LVEF data between the ARNI and ACEI/ARB subgroups at 6 weeks and 4 months of follow-up is presented in Table 6 and Table 7.

For Table 4, Table 5, Table 6 and Table 7: ACEI: angiotensin-converting enzyme inhibitor; ARB: angiotensin II receptor blocker; ARNI: angiotensin receptor–neprilysin inhibitor; LVEF: left ventricular ejection fraction.

### 3.6. Clinical Outcomes in the ARNI and ACEI/ARB Subgroups

During the 1–3-year (695 [456; 996] days) follow-up, one patient (3.3%) in the ARNI group and two (6.7%) patients in the ACEI/ARB group died. In the ACEI/ARB group, three (10%) patients were re-hospitalized due to HF decompensation or reinfarction. The differences were statistically insignificant (Table 8).

## 4. Discussion

Activation of the renin–angiotensin–aldosterone system (RAAS) and increased activity of the sympathetic nervous system are important elements of the pathophysiology of heart failure after myocardial infarction. The RAAS plays a key role in the process of adverse left ventricular remodeling through pro-inflammatory, pro-fibrotic, and hypertrophic effects. Inhibition of this system by ACEI or ARB plays a key role in the pharmacotherapy of HF. The second important system involved in the pathophysiology of HF is the natriuretic system, which is regulated by neprilysin, an enzyme that breaks down natriuretic peptides with vasodilatory, antifibrotic, and diuretic effects. Inhibition of neprilysin (e.g., sacubitril) leads to an increase in the concentration of these beneficial peptides, thus counteracting the action of the RAA and adrenergic systems. Early initiation of adequate pharmacotherapy after AMI, especially in patients with left ventricular dysfunction, is crucial to prevent progression to chronic HFrEF and to limit adverse myocardial remodeling. The current European Society of Cardiology (ESC) guidelines recommend initiating ACEI or ARB therapy as early as possible after MI, especially in patients with reduced LVEF or signs of HF [6].

The results of our work are a summary of the experiences of one center in the use of ARNI in patients with AMI and significant post-infarction left ventricular systolic dysfunction. At a time when ARNI has a well-established position in the current ESC guidelines on the treatment of HF [2,3], its use in patients with AMI (despite evidence of non-inferiority compared to conventional ACEI/ARB-based pharmacotherapy) has not been clearly defined in the current guidelines [6].

Although the retrospective nature of the study, we made every effort to ensure that the impact of ARNI on the parameters we assessed was clear and not confounded by other variables. Therefore, we employed very detailed and extensive inclusion and exclusion criteria to obtain a population of patients who were free from other diseases, who had no previous history of HF, and for whom this was the first episode of coronary artery disease (CAD). We believe that we met these assumptions, and the presented results support this, as the patients had no significant underlying disease burden except for hypertension, diabetes, hyperlipidemia, obesity, and smoking, which are themselves risk factors for CAD. A second important objective was to obtain as similar populations as possible in the ARNI and ACEI/ARB subgroups. Therefore, we employed 1:1 nearest neighbor matching—the obtained subgroups did not differ in terms of clinical characteristics, type of MI, basic laboratory test results, or pharmacotherapy. The only significant variable was a higher frequency of SLGT-2 inhibitor use (*p* = 0.01) and a trend toward a lower LVEF (*p* = 0.076) in the ARNI subgroup. All of these factors ultimately influenced the size of the analyzed group, which consisted of 60 patients, representing only 4.1% of all patients hospitalized with a diagnosis of AMI during the study period.

Both ARNI and ACEI/ARB significantly improved post-infarction left ventricular systolic dysfunction at 6 weeks and 4 months of follow-up. In the context of the discussed results, it should be noted that the ARNI subgroup had a lower baseline LVEF (30% [28; 38]) in a borderline manner than the ACEI/ARB (36% [33; 39]). Importantly, the ACEI/ARB subgroup demonstrated a significantly greater increase in LVEF and relative LVEF compared to the ARNI subgroup at the 6-week follow-up. After 4 months, the differences between the subgroups were blurred; however, ARNI appeared to demonstrate a potential advantage in improving the LVEF, although the difference was not statistically significant. When interpreting the study results, it is important to take into account that the vast majority of patients in the ARNI group (99.7%) received the lowest available dose, which could have attenuated the early treatment effect. Differences in drug titration, tolerability between the two treatment strategies, and more advanced LV dysfunction in the ARNI group may also contribute to the observed temporal pattern of LVEF recovery. Nonetheless, these results may suggest that the clinical benefit of ARNI treatment becomes apparent over the longer term, which requires further observation. In the case of death and rehospitalization during the follow-up period of 695 ([456; 996]) days, single cases were recorded in both subgroups.

Experimental studies in animal models suggest that sacubitril/valsartan may have a range of beneficial cardioprotective effects following myocardial infarction. ARNI has been shown to improve the LVEF, reduce adverse cardiac remodeling, limit myocardial fibrosis, and modulate post-infarction inflammation and arrhythmogenicity. In studies conducted on animal models (pigs, rats, mice), sacubitril/valsartan significantly improves cardiac function and inhibits remodeling by modulating several molecular pathways. These include suppression of the tryptophan–kynurenine axis, reduction of pro-inflammatory cytokines, promotion of macrophage polarization to the M2 phenotype, activation of PI3K/Akt signaling, and inhibition of NLRP3-dependent pyroptosis via the TAK1/JNK pathway [7,8,9,10]. Antiarrhythmic effects of sacubitril/valsartan were also demonstrated in a rabbit MI model, where the drug ameliorated postinfarct heart function impairment and electrophysiologic remodeling, leading to reduced ventricular tachyarrhythmia inducibility by stabilizing the infarct scar and myocardial electrophysiology [11]. Other studies have shown that ARNI reduces collagen synthesis and myocardial fibrosis by inhibiting the synthesis pathways of TGF-β1, Smad proteins, and Wnt/β-catenin-pathway-related proteins [12,13]. Reports from the rat model are reflected in a clinical trial conducted on 73 patients—the results of the research paper of Wang, L. et al. (2024) confirm that early use of sacubitril/valsartan after AMI has a significant benefit and may have a beneficial effect on ventricular remodeling, regulate the expression levels of TGF-β1 and Smad3, inhibit the TGF-β1/Smad3 signaling pathway, improve left ventricular function, and have a beneficial effect on quality of life while maintaining a safety profile [14]. The results of these studies suggest that sacubitril/valsartan may play a multifaceted role in preventing adverse cardiac remodeling post-MI, including anti-fibrotic, anti-inflammatory, and antiarrhythmic effects, mediated through diverse signaling pathways. Notably, many of these processes—particularly the reversal of fibrosis and structural restoration—are time-dependent and do not occur immediately. Therefore, the effects of ARNI therapy on remodeling may not be fully apparent during a short-term follow-up, such as 6 weeks. The early therapeutic impact may be limited by initially low dosing, differences in bioavailability, and the time required to activate intracellular signaling pathways that mediate myocardial repair. Animal models have also shown that the beneficial effects of ARNI on left ventricular structure and function become more evident only after several weeks or even months of treatment, potentially amplifying the benefit observed at the 4-month follow-up. Although these data require further confirmation in clinical trials, they provide a strong biological basis for further testing of ARNI in patients with AMI, particularly those at high risk for left ventricular remodeling.

The benefits of using ARNI in AMI are not limited to experimental studies but are also confirmed in everyday clinical settings among real-world patient populations. In the study by Liu et al. (2024), it was shown that in patients with acute coronary syndrome (ACS) and concomitant reduced LVEF, sacubitril/valsartan treatment and routine treatment led to improvement in the LVEF, NT-proBNP concentration, and left ventricular dimensions, while in the ARNI group, the assessed parameters were significantly improved over those in the routine group [15]. An additional conclusion from the study is that patients with ACS and reduced LVEF may derive greater benefits [15]. Fan, H. et al. (2023), in a group of 78 patients with AMI undergoing PCI, treated for at least 3 months, confirmed the superiority of ARNI over irbesartan in terms of improving cardiac function, preventing ventricular remodeling, and causing a lower incidence of major adverse cardiovascular events (MACE) [16]. The randomized, single-blind, parallel-group, controlled trial performed by Yin, H. et al. (2024) on 142 patients with AMI complicated by moderate to severe mitral regurgitation (MR) treated with ARNI or benazepril showed that early use of ARNI can significantly reduce MR, improve ventricular remodeling, and decrease HF hospitalization compared to ACEI in 12 months of follow-up [17]. In the context of comparisons with ramipril, the clinical trials conducted by Rezq, A. et al. (2021) showed that sacubitril/valsartan may be a safe and effective alternative even in patients with STEMI complicated by cardiogenic shock and that ARNI therapy led to a lower rate of hospitalization due to HF in a 6-month follow-up (18% vs. 38%) [18]. In another study, the same team showed that in patients after STEMI, ARNI additionally has a more beneficial effect on improving LVEF and LV remodeling compared to ramipril, with the same safety profile [19]. Altogether, these clinical studies confirm that in real hospital practice, sacubitril/valsartan can be an effective and safe element of post-infarction therapy, bringing benefits both in terms of echocardiographic parameters and hard endpoints. Importantly, the obtained effects are consistent with previously described mechanisms of action observed in experimental models.

SGLT-2 inhibitors have been shown to reduce the risk of worsening HF and cardiovascular death in patients with HF, regardless of the LVEF or diabetes status [20,21,22,23]. Although they are not yet routinely recommended after AMI, emerging evidence suggests their potential role in early post-AMI management. The EMPACT-MI and EMMY trials demonstrated that early administration of empagliflozin may improve cardiac function, accelerate NT-proBNP reduction, and support reverse remodeling, indicating a possible benefit in preventing heart failure progression in this population [24,25]. Both studies support the hypothesis that SGLT-2 inhibitors may play an important role in early pharmacotherapy after AMI. Among patients with a lower baseline LVEF, we decided to start this therapy more often; as a result, patients who received ARNI were also significantly more likely to be started on SLGT 2 inhibitors (21 vs. 11 patients, *p* = 0.01). In light of the latest reports, a higher proportion of SGLT2 inhibitor use in the ARNI group may have influenced the observed outcomes and confounded the clear attribution of benefit to ARNI therapy alone, but the combination of RAAS inhibitors and SGLT-2 inhibitors in this group of patients is necessary and could not be omitted.

It should be noted that the positive effect of ARNI on LV remodeling was well documented in de novo dilated cardiomyopathy (DCM). The results of the PROVE-HF and EVALUATE-HF studies provide substantial evidence for the beneficial effect of ARNI therapy on left ventricular remodeling in patients with HFrEF. In the PROVE-HF study, 12 months of ARNI therapy were associated with a parallel decrease in NT-proBNP and significant improvements in LVEF and left ventricular volume [26]. In the EVALUATE-HF study, significant improvements in left ventricular structural and functional parameters were observed in the ARNI group compared with enalapril after just 12 weeks [27]. In patients with non-ischemic DCM, earlier ARNI administration was associated with greater LVEF improvement and more effective reverse remodeling [28]. In the case of post-AMI remodeling and scarring in the LV, wall a potent positive ARNI effect is not so important.

In recent years, a number of meta-analyses and systematic reviews assessing the efficacy and safety of early use of ARNI in AMI patients have been published. In the meta-analysis by Liu Y. et al. (2024), which included studies involving patients after percutaneous coronary angioplasty (PCI), it was shown that treatment with sacubitril/valsartan led to a significant reduction in both left ventricular end-diastolic and end-systolic dimensions and volumes, as well as an improvement in ejection fraction (by an average of 3.9 percentage points) [29]. Additionally, an improvement in physical capacity was noted (an increase in the 6-minute walk test [6-WMT] distance by 43 m), as was a decrease in NT-proBNP levels. Importantly, a reduction in the risk of MACE (by 64%), reinfarction (by 46%), and hospitalization for heart failure (by 65%) was also noted without an increase in the risk of renal failure, hyperkalemia, or significant hypotension [29]. Similar results were obtained in the review by Zhou et al. (2022), which showed that ARNI use was associated with a reduction in left ventricular end-diastolic dimensions, as well as with improved exercise tolerance (increase 6-MWT distance by ~48 m) [30]. The risk of cardiovascular events was reduced by 28%, and the risk of rehospitalization for HF by 27%. Although symptomatic hypotension was slightly more frequent in the analysis, cough was less frequent than in the ACEI group. Additionally, ARNI increased the LVEF and decreased the NT-proBNP level, which was better at 6 months and within 3 months of follow-up compared with the control group, but there was no significant difference at the 12-month follow-up [30]. The meta-analysis by Wang, F. et al. (2024) showed a reduction in major adverse cardiovascular and cerebrovascular events (MACCEs) (by 52%) and rehospitalizations (by 64%) and an improvement in the LVEF by an average of 2.86 percentage points [31]. At the same time, no differences in adverse events were found between the study and control groups [31]. In the meta-analysis by Abdullah et al. (2024), a significant reduction in the risk of hospitalization for HF (by 21%) and MACE (by 16%) was demonstrated, as well as a decrease in NT-proBNP levels [32]. Although the improvement in the LVEF was greater in the control group compared with ARNI, the observed differences suggest a therapeutic benefit in selected patients [32]. The study conducted by She, J. et al. (2021) showed that patients who received ARNI had significantly lower rates of the composite cardiovascular outcome than ACEI and ARB patients [33]. Patients who received ARNI had lower rates of cardiovascular deaths, and the subanalysis showed that the greatest benefit was experienced by patients younger than 60 years of age and with an LVEF of less than 40% [33].

Available systematic reviews and meta-analyses suggest that early use of ARNI after MI can lead to significant improvements in cardiac remodeling and echocardiographic parameters. ARNI therapy can reduce the risk of cardiovascular events, including hospitalization for HF, and this drug has a favorable safety profile, with well-tolerated hypotension and a lower incidence of adverse events typical of ACEIs, such as cough. These results are consistent with the observations from our study, indicating a potential benefit of early initiation of ARNI after MI, especially in patients with a reduced LVEF. Although the results are promising, further large randomized trials with long-term follow-up are needed to clearly assess the effect of this therapy on survival and progression to HF.

Respective limitations secondary to the retrospective character of the study should be addressed when interpreting the results. First, the results may reflect local practice because the study was conducted at a single center. The small group of patients subjected to the final analysis resulted from very detailed inclusion and exclusion criteria, which we consider to be a strong point of our work; however, a small sample size may influence the nature of the cohort and limit some analyses. Certain limitations arose from the retrospective nature of the study. An important inclusion criterion was participation in the “KOS-zawał” program, which enabled follow-up of patients in the hospital outpatient clinic; however, in some patients, there were missing data in the medical records, which resulted in a reduction in the 4-month follow-up group. It should be emphasized that during the 6-week follow-up period, the doses of ACEI/ARB and ARNI were not escalated, which is associated with failure to achieve the optimal therapeutic effect, especially in the ARNI group, where 99.7% of patients started treatment with the lowest dose, which represents a methodological limitation of the study. Moreover, different ACEI/ARB treatments were administered. An additional confounding factor may be the effect of SGLT-2 inhibitors on adverse remodeling; these were more frequently used in the ARNI group. We believe that, despite its limitations, our study provides important conclusions about the use of ARNI in patients with AMI in real-life experience and shows a significant trend in which patients may benefit most from ARNI use. Finally, our results confirm the findings from numerous clinical trials, meta-analyses, and reviews, and the molecular basis is confirmed by experiments conducted in animal models.

## 5. Conclusions

Our current experience in ARNI therapy after AMI is promising, but it is limited to a small group of patients with severe impairment of LV systolic function. Regardless of the significant improvement in the baseline LVEF observed in AMI patients receiving both ACE/ARB and ARNI in 6 weeks and 4 months of follow-up, the values of absolute and relative LVEF increases were higher in subjects treated with ACEI/ARB. Importantly, after 4 months of therapy, the differences between the groups were blurred; however, ARNI appeared to demonstrate a potential advantage in improving the LVEF, although the difference was not statistically significant. There were no differences in cardiovascular outcomes between the ARNI and ACEI/ARB populations. The use of ARNI early in the setting of AMI is becoming more widespread; nevertheless, additional research is necessary.

## Figures and Tables

**Table 1:** Inclusion and exclusion criteria.

Inclusion Criteria	Exclusion Criteria
(1) Age ≥ 18 years	(1) A history of previous myocardial infarction or coronary revascularization
(2) First diagnosis of MI	(2) A history of HF or angioedema
(3) Percutaneous revascularization of the infarct-related coronary artery and complete revascularization during current hospitalization	(3) Co-occurrence of another heart disease affecting the left ventricular ejection fraction
(4) Post-infarction left ventricular systolic dysfunction (LVEF ≤ 40%)	(4) Severe hepatic or renal dysfunction
(5) Patients enrolled into coordinated specialist care after a MI (“KOS zawał” program)	(5) Coexisting severe diseases of the immune, hematopoietic, or respiratory systems or systemic diseases
	(6) Presence of a malignant tumor
	(7) Death of a patient during hospitalization
	(8) Incomplete clinical data in the medical records

HF—heart failure; LVEF—left ventricular ejection fraction; MI—myocardial infarction.

**Table 2:** Baseline characteristics of the subgroups before and after matching.

Variable	Pre-Match *n* (%) or Mean ± SD or Median (1–3 Quartiles)			Post-Match *n* (%) or Mean ± SD or Median (1–3 Quartiles)		
	ARNI Subgroup *n* = 30	ACEI/ARB Subgroup *n* = 46	Std. Mean Diff.	ARNI Subgroup *n* = 30	ACEI/ARB Subgroup *n* = 30	Std. Mean Diff.
Age (years)	58 ± 10	65 ± 10	0.708	58 ± 10	62 ± 10	0.388
Men (%)	27 (90%)	31 (67.4%)	0.567	27 (90%)	26 (86.7%)	0.102
LVEF (%)	30 (28; 38)	38 (33; 40)	0.704	30 (28; 38)	36 (33; 39)	0.451

ACEI: angiotensin-converting enzyme inhibitor; ARB: angiotensin II receptor blocker; ARNI: angiotensin receptor–neprilysin inhibitor; LVEF: left ventricular ejection fraction, Std. Mean Diff.: standardized mean difference.

**Table 3:** The clinical characteristics of the ARNI and ACEI/ARB subgroups.

Factor	ARNI Subgroup *n* = 30 *n* (%) or Mean ± SD or Median (1–3 Quartile)	ACEI/ARB Subgroup *n* = 30 *n* (%) or Mean ± SD or Median (1–3 Quartile)	*p* Value
Basic characteristic			
Age (years)	58 ± 10	62 ± 10	0.14
Sex-men (*n*%)	27 (90%)	26 (86.7%)	0.69
BMI (kg/m^2^)	29 ± 4.5	28.7 ± 4.2	0.8
Type of myocardial infarction			
STEMI (*n*%)	24 (80%)	23 (76.7%)	0.756
Anterior wall (*n*%)	17 (56.7%)	13 (43.3%)	-
Inferior wall (*n*%)	4 (13.3%)	6 (20%)	-
Anterior and lateral wall (*n*%)	3 (10%)	3 (10%)	-
Inferior and lateral wall (*n*%)	0 (0%)	1 (3.3%)	-
NSTEMI (*n*%)	6 (20%)	7 (23.3%)	0.756
Laboratory tests			
Serum creatinine level (mg/dL)	0.83 (0.75; 1.03)	1.0 ± 0.25	0.1
eGFR (mL/min/1.73)	66.5 ± 12.8	67.8 ± 14.98	0.819
eGFR < 60 (mL/min/1.73)	2 (6.7%)	5 (16.7%)	0.232
Troponin max. level (ng/mL)	3.46 (0.64; 5.12)	1.03 (0.15; 3.48)	0.085
Hemoglobin level (g/dL)	14.71 ± 1.33	14.86 ± 1.55	0.681
Echocardiographic parameters			
LVEF (%)	30 (28; 38)	36 (33; 39)	0.076
LVEDD (mm)	52 (50; 59)	53.18 ± 6.85	0.212
LVESD (mm)	40.12 ± 10.38	37.25 ± 7.8	0.257
LVIVSd (mm)	11.27 ± 1.71	12.1 ± 1.8	0.085
LVPWd (mm)	9.61 ± 1.47	10 (9; 11)	0.204
LAarea (cm^2^)	21.86 ± 6.69	22.1 ± 4.57	0.927
LAwidth (mm)	40.72 ± 5.8	40 (38; 44.5)	0.67
Mitral regurgitation (II or III) (*n*%)	3 (10%)	2 (6.7%)	1.0
Tricuspid regurgitation (II or III) (*n*%)	4 (13.3%)	1 (3.3%)	0.353
Aortic regurgitation (*n*%)	0 0 (%)	2 (6.7%)	0.492
Aortic stenosis (*n*%)	0 0 (%)	1 (3.3%)	1.0
Concomitant diseases			
Atrial fibrillation (*n*%)	6 (20%)	7 (23.3%)	0.756
Chronic kidney disease (*n*%)	1 (3.3%)	2 (6.7%)	1.0
Hypertension (*n*%)	21 (70%)	23 (76.7%)	0.563
Diabetes (*n*%)	6 (20%)	12 (40%)	0.093
Lipid disorders (*n*%)	23 (76.7%)	25 (83.3%)	0.522
Smoke history (*n*%)	18 (60%)	18 (60%)	1.0
History of stroke (*n*%)	0 0 (%)	1 (3.3%)	1.0
Asthma (*n*%)	0 0 (%)	2 (6.7%)	0.492
Chronic obstructive pulmonary disease (*n*%)	1 (3.3%)	3 (10%)	0.612
Medicinal treatment			
ARNI (*n*%)	24/26 mg – 29 (99.7%) 49/51 mg – 1 (0.3%)	-	
ACEI/ARB (*n*%)	-	ACEI – 28 (93.3%) ARB – 2 (6.7%)	
SGLT-2 inhibitor (*n*%)	21 (70%)	11 (36.7%)	0.01
Beta-blocker (*n*%)	28 (93.3%)	25 (83.3%)	0.232
Mineralocorticoid-receptor antagonist (*n*%)	26 (86.7%)	21 (70%)	0.12
Loop diuretic (*n*%)	15 (50%)	8 (26.7%)	0.065
Calcium channel blockers (*n*%)	1 (3.3%)	5 (16.7%)	0.195
Statin (*n*%)	30 (100%)	30 (100%)	1.0
Ezetimibe (*n*%)	11 (36.7%)	7 (23.3%)	0.264
Fibrate (*n*%)	0 (%)	2 (6.7%)	0.492
GLP-1 receptor agonists (*n*%)	1 (3.3%)	2 (6.7%)	1.0
Metformin (*n*%)	2 (6.7%)	8 (26.7%)	0.08
Sulfonylureas (*n*%)	0 (%)	4 (13.3%)	0.112
Insulin (*n*%)	1 (3.3%)	1 (3.3%)	1.0
Acetylsalicylic acid (*n*%)	30 (100%)	30 (100%)	1.0
P2Y12 inhibitors (*n*%)	30 (100%)	30 (100%)	1.0
NOAC (*n*%)	6 (20%)	7 (23.3%)	0.756

ARNI: angiotensin receptor–neprilysin inhibitor; ACEI: angiotensin-converting enzyme inhibitor; ARB: angiotensin II receptor blocker; STEMI: ST-elevation myocardial infarction; NSTEMI: non-ST-elevation myocardial infarction; BMI: body mass index; LVEF: left ventricular ejection fraction; LVEDD: left ventricular end-diastolic diameter; LVESD: left ventricular end-systolic diameter; LVIVSd: left ventricular interventricular septal diameter (at end-diastole); LVPWd: left ventricular posterior wall diameter (at end-diastole); LAarea: left atrial area; LAwidth: left atrial width; SGLT-2: sodium-glucose co-transporter 2; GLP-1: glucagon-like peptide-1; P2Y12: P2Y12 receptor (platelet ADP receptor subtype involved in platelet aggregation); NOAC: non-vitamin K antagonist oral anticoagulants.

**Table 4:** Comparison of the baseline and 6-week follow-up values of the LVEF in the ARNI and ACEI/ARB subgroups.

Subgroup	Baseline LVEF (%) Median (1–3 Quartiles)	LVEF (%) in 6 Weeks Median (1–3 Quartiles)	*p* Value
ARNI *n* = 29	30 (27.3; 38)	37 (30; 43)	0.0008
ACEI/ARB *n* = 30	36 (33; 39)	45 (42; 52)	<0.0001

**Table 5:** Comparison of the baseline and 4-month follow-up values of the LVEF in the ARNI and ACEI/ARB subgroups.

Subgroup	Baseline LVEF (%) *n* (%) or Mean ± SD	LVEF (%) in 4 Months *n* (%) or Mean ± SD	*p* Value
ARNI *n* = 20	30.5 ± 5.9	40.2 ± 7.8	0.0001
ACEI/ARB *n* = 7	34.9 ± 7.3	44.6 ± 10.9	0.018

**Table 6:** Comparison of the LVEF data in the ARNI and ACEI/ARB subgroups in the context of the 6-week follow-up.

Factor	ARNI Subgroup *n* = 29 Median (1–3 Quartiles)	ACEI/ARB Subgroup *n* = 30 Median (1–3 Quartiles)	*p* Value
Baseline LVEF (%)	30 (27.3; 38)	36 (33; 39)	0.072
LVEF (%) in 6 weeks	37 (30; 43)	45 (42; 52)	0.003
ΔLVEF (%)	6 (2; 10.25)	10 (6; 12)	0.018
Relative ΔLVEF (%)	17.5 (7; 31.9)	30 (15.4; 40)	0.047

**Table 7:** Comparison of the LVEF data in the ARNI and ACEI/ARB subgroups in the context of the 4-month follow-up.

Factor	ARNI Subgroup *n* = 20 *n* (%) or Mean ± SD	ACEI/ARB Subgroup *n* = 7 *n* (%) or Mean ± SD	*p* Value
Baseline LVEF (%)	30.5 ± 5.9	34.85 ± 7.27	0.073
LVEF (%) in 4-months	40.2 ± 7.7	44.6 ± 10.9	0.025
ΔLVEF (%)	9.65 ± 8.6	9.71 ± 8.01	0.986
Relative ΔLVEF (%)	35.6 ± 34.07	30.11 ±27.8	0.705

**Table 8:** Comparison of clinical outcomes in the ARNI and ACEI/ARB subgroups.

Clinical Outcome	ARNI Subgroup *n* = 30 *n* (%) or Mean ± SD or Median (1–3 Quartiles)	ACEI/ARB Subgroup *n* = 30 *n* (%) or Mean ± SD or Median (1–3 Quartiles)	*p* Value
Deaths (*n*%)	1 (3.3%)	2 (6.7%)	1.0
Number of rehospitalizations (*n*%)	0	3 (10%)	0.24

ACEI: angiotensin-converting enzyme inhibitor; ARB: angiotensin II receptor blocker; ARNI: angiotensin receptor–neprilysin inhibitor.

## Data Availability

The data presented in this study are available from the corresponding author upon request. The data are not publicly available due to the confidentiality of the research.

## Abbreviations

The following abbreviations are used in this manuscript:

6-WMT	6-minute walk test
ACS	acute coronary syndrome
AMI	acute myocardial infarction
ARNI	angiotensin receptor–neprilysin inhibitor
CABG	coronary artery bypass grafting
CAD	coronary artery disease
DCM	dilated cardiomyopathy
ESC	European Society of Cardiology
HF	heart failure
HFrEF	heart failure with reduced ejection fraction
LAV	left atrial volume
LVEF	left ventricular ejection fraction
MACE	major adverse cardiovascular events
MACCE	major adverse cardiovascular and cerebrovascular events
MI	myocardial infarction
MR	mitral regurgitation
MRA	mineralocorticoid receptor antagonist
NN	nearest neighbor
RAAS	renin–angiotensin–aldosterone system
SGLT-2	Inhibitors sodium-glucose co-transporter 2 inhibitors
STEMI	ST-elevation myocardial infarction

## References

[1] McMurray J.J., Packer M., Desai A.S., Gong J., Lefkowitz M.P., Rizkala A.R., Rouleau J.L., Shi V.C., Solomon S.D., Swedberg K. (2014). PARADIGM-HF Investigators and Committees. Angiotensin-neprilysin inhibition versus enalapril in heart failure. N. Engl. J. Med..

[2] McDonagh T.A., Metra M., Adamo M., Gardner R.S., Baumbach A., Böhm M., Burri H., Butler J., Čelutkienė J., ESC Scientific Document Group (2021). 2021 ESC Guidelines for the diagnosis and treatment of acute and chronic heart failure. Eur. Heart J..

[3] McDonagh T.A., Metra M., Adamo M., Gardner R.S., Baumbach A., Böhm M., Burri H., Butler J., Čelutkienė J., ESC Scientific Document Group (2023). 2023 Focused Update of the 2021 ESC Guidelines for the diagnosis and treatment of acute and chronic heart failure. Eur. Heart J..

[4] Pfeffer M.A., Claggett B., Lewis E.F., Granger C.B., Køber L., Maggioni A.P., Mann D.L., McMurray J.J., Rouleau J.-L., Solomon S.D. (2021). PARADISE-MI Investigators and Committees. Angiotensin Receptor-Neprilysin Inhibition in Acute Myocardial Infarction. N. Engl. J. Med..

[5] Shah A.M., Claggett B., Prasad N., Li G., Volquez M., Jering K., Cikes M., Kovacs A., Mullens W., Nicolau J.C. (2022). Impact of Sacubitril/Valsartan Compared With Ramipril on Cardiac Structure and Function After Acute Myocardial Infarction: The PARADISE-MI Echocardiographic Substudy. Circulation.

[6] Byrne R.A., Rossello X., Coughlan J.J., Ibanez B., Barbato E., Berry C., Chieffo A., Claeys M.J., Dan G.-A., Members of the Task Force for the 2023 ESC Guidelines for the management of acute coronary syndromes (2023). ESC Scientific Document Group. 2023 ESC Guidelines for the management of acute coronary syndromes. Eur. Heart J..

[7] Martínez-Falguera D., Aranyó J., Teis A., Ferrer-Curriu G., Monguió-Tortajada M., Fadeuilhe E., Rodríguez-Leor O., Díaz-Güemes I., Roura S., Villuendas R. (2024). Antiarrhythmic and Anti-Inflammatory Effects of Sacubitril/Valsartan on Post-Myocardial Infarction Scar. Circ. Arrhythm. Electrophysiol..

[8] Gan J., Wang Y., Deng Y., Zhang J., Wang S., Jiang X., Guo M., Song L. (2024). Sacubitril/valsartan ameliorates cardiac function and ventricular remodeling in CHF rats via the inhibition of the tryptophan/kynurenine metabolism and inflammation. Sci. Rep..

[9] Jin N., Qiu Y., Zhang K., Fang Y., Qu S., Zhu L., Li H., Nie B. (2024). Sacubitril/valsartan alleviates myocardial infarction-induced inflammation in *mice* by promoting M2 macrophage polarisation via regulation of PI3K/Akt pathway. Acta Cardiol..

[10] Shen J., Fan Z., Sun G., Qi G. (2021). Sacubitril/valsartan (LCZ696) reduces myocardial injury following myocardial infarction by inhibiting NLRP3-induced pyroptosis via the TAK1/JNK signaling pathway. Mol. Med. Rep..

[11] Chang P.C., Wo H.T., Lee H.L., Lin S.-F., Chu Y., Wen M.-S., Chou C.-C. (2020). Sacubitril/Valsartan Therapy Ameliorates Ventricular Tachyarrhythmia Inducibility in a *Rabbit* Myocardial Infarction Model. J. Card. Fail..

[12] Wu M., Guo Y., Wu Y., Xu K., Lin L. (2021). Protective Effects of Sacubitril/Valsartan on Cardiac Fibrosis and Function in Rats With Experimental Myocardial Infarction Involves Inhibition of Collagen Synthesis by Myocardial Fibroblasts Through Downregulating TGF-β1/Smads Pathway. Front. Pharmacol..

[13] Liu J., Zheng X., Zhang C., Bu P. (2021). Lcz696 Alleviates Myocardial Fibrosis After Myocardial Infarction Through the sFRP-1/Wnt/β-Catenin Signaling Pathway. Front. Pharmacol..

[14] Wang L., Zhang Y., Xue J., Da Y., Gao Y., Sun Y., Zhou S. (2024). Early Application of Sacubitril Valsartan Sodium After Acute Myocardial Infarction and its Influence on Ventricular Remodeling and TGF-β1/Smad3 Signaling Pathway. Altern. Ther. Health Med..

[15] Liu H., Su Y., Shen J., Jiao Y., Li Y., Liu B., Hou X., Jin Q., Chen Y., Sun Z. (2024). Improved heart function and cardiac remodelling following sacubitril/valsartan in acute coronary syndrome with HF. ESC Heart Fail..

[16] Fan H., Wang Y., Wang X., Dong X., Shao X., Yang F. (2023). Effect of Emergency Percutaneous Coronary Intervention Combined with Sacubitril and Valsartan on the Cardiac Prognosis in Patients with Acute Myocardial Infarction. Int. J. Gen. Med..

[17] Yin H., Ma L., Zhou Y., Tang X., Li R., Zhou Y., Shi J., Zhang J. (2024). Efficacy of early administration of sacubitril/valsartan after coronary artery revascularization in patients with acute myocardial infarction complicated by moderate-to-severe mitral regurgitation: A randomized controlled trial. Heart Vessel..

[18] Rezq A., Saad M., El Nozahi M. (2021). Sacubitril/valsartan versus ramipril in patients with ST-segment Elevation Myocardial Infarction and cardiogenic SHOCK (SAVE-SHOCK): A pilot randomized controlled trial. Am. J. Cardiovasc. Dis..

[19] Rezq A., Saad M., El Nozahi M. (2021). Comparison of the Efficacy and Safety of Sacubitril/Valsartan versus Ramipril in Patients With ST-Segment Elevation Myocardial Infarction. Am. J. Cardiol..

[20] McMurray J.V., Solomon S.D., Inzucchi S.E., Køber L., Kosiborod M.N., Martinez F.A., Ponikowski P., Sabatine M.S., Anand I.S., Bělohlávek J. (2019). Dapagliflozin in patients with heart failure and reduced ejection fraction. N. Engl. J. Med..

[21] Packer M., Anker S.D., Butler J., Filippatos G., Pocock S.J., Carson P., Januzzi J., Verma S., Tsutsui H., Brueckmann M. (2020). Cardiovascular and renal outcomes with empagliflozin in heart failure. N. Engl. J. Med..

[22] Anker S.D., Butler J., Filippatos G., Ferreira J.P., Bocchi E., Böhm M., La Rocca H.-P.B., Choi D.-J., Chopra V., Valenzuela E. (2021). Empagliflozin in heart failure with a preserved ejection fraction. N. Engl. J. Med..

[23] Solomon S.D., McMurray J.V., Claggett B., de Boer R.A., DeMets D., Hernandez A.F., Inzucchi S.E., Kosiborod M.N., Lam C.S., Martinez F. (2022). Dapagliflozin in heart failure with mildly reduced or preserved ejection fraction. N. Engl. J. Med..

[24] Butler J., Jones W.S., Udell J.A., Anker S.D., Petrie M.C., Harrington J., Mattheus M., Zwiener I., Bahit M.C., Bauersachs J. (2024). Empagliflozin after Acute Myocardial Infarction. N. Engl. J. Med..

[25] Von Lewinski D., Kolesnik E., Tripolt N.J., Pferschy P.N., Benedikt M., Wallner M., Alber H., Berger R., Lichtenauer M., Saely C.H. (2022). Empagliflozin in acute myocardial infarction: The EMMY trial. Eur. Heart J..

[26] Januzzi J.J., Prescott M.F., Butler J., Felker G.M., Maisel A.S., McCague K., Camacho A., Piña I.L., Rocha R.A., Shah A.M. (2019). PROVE-HF Investigators. Association of Change in N-Terminal Pro-B-Type Natriuretic Peptide Following Initiation of Sacubitril-Valsartan Treatment With Cardiac Structure and Function in Patients With Heart Failure With Reduced Ejection Fraction. JAMA.

[27] Desai A.S., Solomon S.D., Shah A.M., Claggett B.L., Fang J.C., Izzo J., McCague K., Abbas C.A., Rocha R., Mitchell G.F. (2019). EVALUATE-HF Investigators. Effect of Sacubitril-Valsartan vs Enalapril on Aortic Stiffness in Patients With Heart Failure and Reduced Ejection Fraction: A Randomized Clinical Trial. JAMA.

[28] Kim H.M., Kim K.H., Park J.S., Oh B.-H. (2021). Beneficial Effect of Left Ventricular Remodeling after Early Change of Sacubitril/Valsartan in Patients with Nonischemic Dilated Cardiomyopathy. Medicina.

[29] Liu Y., Sun Y., Dai W. (2024). Effect of sacubitril-valsartan on left ventricular remodeling in patients with acute myocardial infarction after primary percutaneous coronary intervention: A systematic review and meta-analysis. Front. Pharmacol..

[30] Zhou X., Zhu H., Zheng Y., Tan X., Tong X. (2022). A systematic review and meta-analysis of sacubitril-valsartan in the treatment of ventricular remodeling in patients with heart failure after acute myocardial infarction. Front. Cardiovasc. Med..

[31] Wang F., Li C., Zhang X. (2024). Sacubitril/valsartan improves the prognosis of acute myocardial infarction: A meta-analysis. Coron. Artery Dis..

[32] Abdullah, Rashid M., Soto C.J., Virk G.S., Mekowulu F.C., Chaudhari S.S., Batool S., Usama M. (2024). The Safety and Efficacy of the Early Use of Sacubitril/Valsartan After Acute Myocardial Infarction: A Meta-Analysis of Randomized Controlled Trials. Cureus.

[33] She J., Lou B., Liu H., Zhou B., Jiang G.T., Luo Y., Wu H., Wang C., Yuan Z. (2021). ARNI versus ACEI/ARB in Reducing Cardiovascular Outcomes after Myocardial Infarction. ESC Heart Fail..

